# Triterpenic Acids Present in Hawthorn Lower Plasma Cholesterol by Inhibiting Intestinal ACAT Activity in Hamsters

**DOI:** 10.1093/ecam/nep007

**Published:** 2010-10-19

**Authors:** Yuguang Lin, Mario A. Vermeer, Elke A. Trautwein

**Affiliations:** Unilever Food and Health Research Institute, Unilever R&D Vlaardingen, P.O. Box 114, 3130 AC Vlaardingen, The Netherlands

## Abstract

Hawthorn (*Crataegus pinnatifida*) is an edible fruit used in traditional Chinese medicine to lower plasma lipids. This study explored lipid-lowering compounds and underlying mechanisms of action of hawthorn. Hawthorn powder extracts inhibited acylCoA:cholesterol acyltransferase (ACAT) activity in Caco-2 cells. The inhibitory activity was positively associated with triterpenic acid (i.e., oleanolic acid (OA) and ursolic acid (UA)) contents in the extracts. Cholesterol lowering effects of hawthorn and its potential additive effect in combination with plant sterol esters (PSE) were further studied in hamsters. Animals were fed a semi-synthetic diet containing 0.08% (w/w) cholesterol (control) or the same diet supplemented with (i) 0.37% hawthorn dichloromethane extract, (ii) 0.24% PSE, (iii) hawthorn dichloromethane extract (0.37%) plus PSE (0.24%) or (iv) OA/UA mixture (0.01%) for 4 weeks. Compared to the control diet, hawthorn, PSE, hawthorn plus PSE and OA/UA significantly lowered plasma non-HDL (VLDL + LDL) cholesterol concentrations by 8%, 9%, 21% and 6% and decreased hepatic cholesterol ester content by 9%, 23%, 46% and 22%, respectively. The cholesterol lowering effects of these ingredients were conversely associated with their capacities in increasing fecal neutral sterol excretion. In conclusion, OA and UA are responsible for the cholesterol lowering effect of hawthorn by inhibiting intestinal ACAT activity. In addition, hawthorn and particularly its bioactive compounds (OA and UA) enhanced the cholesterol lowering effect of plant sterols.

## 1. Introduction

Hawthorn is a berry-like fruit of trees from the species of *Crataegus*, which has been documented as a food in China as early as the seventh millennium before Christ [1]. Hawthorn is also one of the commonly used remedies in traditional Chinese medicine (TCM). The history and traditional uses of (10 g/day) dry hawthorn in TCM is to relieve fullness that follows overeating or consumption of a fatty meal. During the last decades hawthorn has received much attention because of its potential to reduce plasma cholesterol and triacylglycerol (TAG) concentrations [2–9] and to treat heart arrhythmia [10–12]. However, the scientific evidence of these beneficial effects of hawthorn still needs to be further substantiated, including identification of its bioactive compounds and the underlying mechanisms of action.

There are different species of *Crataegus*. In China, *Crataegus pinnatifida* is predominantly used as TCM remedy [2, 6, 8], while *C. monogyna* and *C. oxyacantha* are commonly used in Europe for the treatment of heart failure [10, 11]. Recently, Zhang et al. [3] reported that an aqueous ethanolic extract of hawthorn (*C. pinnatifida*) could inhibit intestinal acylCoA:cholesterol acyltransferase (ACAT) activity in hamsters. However, bioactive compounds responsible for the inhibition of ACAT in the hawthorn extract have not yet been identified. Hawthorn is rich in triterpenic acids (e.g., oleanolic acid (OA) and ursolic acid (UA)) and polyphenols (e.g., epicatechin, procyanidin B2, procyanidin B5, procyanidin C1, hyperoside, isoquercitrin and chlorogenic acid) [13]. In the present study, we aimed to identify ACAT inhibiting compounds in hawthorn. Initial tests in Caco-2 cells, a human intestinal cell line, have shown that OA and UA are the bioactive compounds present in hawthorn that inhibit ACAT activity. To further confirm these *in vitro* findings, we conducted a study in hamsters to investigate the cholesterol lowering effect of a hawthorn extract and a mixture of OA/UA resembling their concentrations found in hawthorn. Since hawthorn would exert its cholesterol lowering effect via a mechanism different from that of plant sterols [14], we further explored in this study whether the combination of plant sterols esters and hawthorn extract would have an additive cholesterol lowering effect.

## 2. Materials and Methods

Caco-2 cells were obtained from the ATCC (American Type Culture Collection), Rockville, MD, USA. RPMI 1640 and Dulbecco's Modified Eagle's Medium (DMEM), penicillin, streptomycin and fetal calf serum (FCS) were purchased from Gibco, Life Technologies Ltd, Paisley, UK. Cell culture plates and flasks were supplied by Corning, Cambridge, MA, USA. D l-Mevalonic acid lactone was acquired from Fluka, Buchs, Switzerland. [9,10-^3^H]oleic acid and [4-^14^C]cholesteryl-oleate were purchased from Amersham, Buckinghamshire, UK. Oleic acid, silicic acid, OA and UA were purchased from Sigma-Aldrich, Zwijndrecht, NL. All organic solutions were obtained from Merck, Amsterdam, NL. One batch of dried hawthorn (*C. pinnatifida*) fruit was obtained from a TCM pharmacy, Rotterdam, NL. Other chemicals were obtained in their commercially available highest purity grade.

### 2.1. Preparation of Hawthorn Extracts

Dried hawthorn fruit was milled to a powder using a grinder. The same batch was used for the *in vitro* experiments and the animal study. For the *in vitro* experiments 20 g of dry hawthorn fruit powder was extracted with 100 mL dichloromethane, ethylacetate, acetone, ethanol or heptane, respectively, at room temperature for 1.5 h. The obtained extracts were dried under nitrogen flow. The yield of these extractions and their OA and UA contents are presented in Table 1.

For the animal study, 425 g of dried hawthorn fruit powder was extracted first with ethanol (2 L) for 12 h at 80°C using a Soxhlet extractor. The ethanol extract was then dried using a vacuum rotary evaporator to obtain a raw ethanol extract. This raw extract was subsequently extracted manually with 1 L dichloromethane under room temperature and the generated extract was dried in order to obtain the hawthorn dichloromethane extract.

### 2.2. Analysis and Quantification of OA and UA in Hawthorn Extracts

The composition of the hawthorn extracts was analyzed using reversed-phase high-performance liquid chromatography (HPLC). The HPLC was carried out on a HP 1090 instrument (Agilent Technologies, Wilmington, DE, USA) equipped with a Supelcosil LC-18-T column (15 cm × 4.6 mm i.d., Supelco, Bellefonte, PA, USA). The hawthorn extracts (100 mg) were dissolved in DMSO (1 mL) and 10 *μ*L of the solutions were injected onto the column. The mobile phase was a mixture of 15% H_2_O, 85% methanol and 0.15% acetic acid (v/v) with a flow rate of 1 mL/min. The effluent was monitored using a UV detector at 210 nm. Identification of OA and UA peaks was based on retention time as comparing with those of pure OA and UA (external standards) under the same conditions.

Further identification and quantification of OA and UA in the hawthorn dichloromethane extract used for the animal study was conducted by using gas chromatography mass spectrometry (GC–MS). GC–MS analysis was performed using an Agilent 6890 Series GC and Micro Mass GCT (Time of Flight Mass Spectrometer, JAS, Eindhoven, NL). The GC analysis was performed with a DB-5MS 15 m × 0.25 mm (*d* = 0.1 *μ*m) column (JAS, Eindhoven, NL). OA/UA containing samples (after derivatization with *N,O*-*bis*(trimethylsilyl)trifluoroacetamide (BSTFA) were dissolved in hexane (*∼*25–30 ng/*μ*L). The flow rate of helium as mobile phase was 1.0 mL/min. Oven temperature program was 1 min at 60°C and then the temperature increased to 310°C (increase 10°C/min) and from 310°C to 325°C (increase 20°C/min) and maintained at 325°C for 25.3 min. Mass spectra were acquired in EI mode using interface temperature of 325°C. The mass spectrometer was operated in positive mode at 70 eV. The scanning range was *m*/*z* 29–650. For quantification of UA, ions 203.1970, 482.4397 and 585.4684 were used. For quantification of OA, ions 203.1961, 482.4407 and 585.4701 were used.

### 2.3. In Vitro Study

#### 2.3.1. Cell Culture

Caco-2 cells were routinely cultured in 75 cm^2^ culture flasks with DMEM supplemented with 20% (v/v) FCS, 10 IU/ml penicillin and 10 *μ*g/mL streptomycin (culture medium). Cells were incubated at 37°C, 5% CO_2_. The medium was refreshed every 2-3 days. Once the cells were confluent, they were passaged with a split ratio of 1 : 4. In experiments, cells were seeded to 24-well plates and cultured for 6 days before they were exposed to the test compounds or extracts. Individual test compounds and extracts were dissolved in dimethylsulfoxide (DMSO). Corresponding amounts of these DMSO solutions (about 50 *μ*g each extract per 10 *μ*L DMSO) were added to cell culture media (1 mL) to have final concentrations as indicated (see Results section, Table 2). Medium DMSO concentration was the same for all treatments and did not exceed 1% (v/v). DMSO concentration up to at least 2% in the medium had no effect on ACAT activity (data not shown). Cells were cultured in these conditioned media for 24 h. Viability of cells was assessed with an ATP assay by using the ViaLight Plus kit LT07-121 (Lonza, Bath, UK) as described previously [15].

#### 2.3.2. ACAT Activity in Caco-2 Cells

Four hours before the end of the overnight cellular incubation with the test compounds or extracts, [9,10-^3^H]oleic acid dissolved in DMEM containing fatty acid-free human serum albumin was added to each well (1 *μ*Ci/well, 62.5 *μ*M final concentration in medium) to monitor synthesis of cholesterol esters. Incubation was ended by transferring the cell culture plates on ice and then the medium was removed. The cells were washed twice with ice-cold phosphate-buffered saline (PBS) and the lipids were extracted twice with isopropyl alcohol : heptane (4 : 1 v/v). The lipid extract was dried under nitrogen flow. The sample was then dissolved in 0.5 mL hexane : ethyl acetate (49 : 1 v/v). The cholesterol esters were isolated from the extracted lipids using a silica column (prepared in a Pasteur's pipette filled with silica) which was eluted four times with 1 mL elution solvent as described by Chautan et al. [16]. The radioactivity of the [^3^H]-labelled cholesterol esters was counted by using a liquid scintillation counter (Packard Tri-Carb Model 1900CA, Downers Grove, USA) after adding 7 mL scintillation cocktail.

### 2.4. Animal Study

This animal study was conducted at the Erasmus Animal Experimental Center, Erasmus University, Rotterdam, NL. The study process was in conformity with the National Research Council Guide for the Care and Use of Laboratory Animals and Public Health Service Policy on Human Care and Use of Laboratory Animals. The study protocol was approved by the ethical committee of Erasmus University, Rotterdam, NL.

#### 2.4.1. Animals

Male Lakeview Golden (LVG) Syrian hamsters (*Mesocricetus auratus*) with a body weight of approximately 75 g were obtained from Charles River Laboratories, Inc., Wilmington, MA, USA. After 1 week of acclimatization, hamsters were randomly allocated to five groups of 20 animals each, based on their body weight. The hamsters were individually housed in Macrolon II cages with a layer of sawdust as bedding. Animals were kept in an environmentally controlled room (temperature 22–25°C and relative humidity *∼*55%) with a 12 h light–dark rhythm (lights off 6:00–18:00 h). Throughout the study, the animals had free access to food and drinking water. Clinical observations were routinely done and 24 h food consumption and body weight gain were measured weekly during the experimental period.

#### 2.4.2. Test Compounds

In the *in vitro* tests, the dichloromethane extract of hawthorn was shown to have the strongest ACAT-inhibitory activity amongst the extracts tested (Table 2). Therefore, the dichloromethane extract (carried out in large scale) was used in the hamster study. The dichloromethane extract contained 0.4% OA and 2.6% UA as measured by HPLC. Plant sterol esters (PSE) were prepared by esterifying soy plant sterols with fatty acids from sunflower oil (esterification degree of >92%) (Unilever Research and Development, Vlaardingen, NL). The soy plant sterol composition was beta-sitosterol 46.7%, beta-sitostanol 1%, campesterol 26.9%, stigmasterol 18.3%, brassicasterol 2.7% and other plant sterols 4.4%.

#### 2.4.3. Diets

During the acclimating period, hamsters were fed a semi-purified diet based on the AIN-93 rodent diet [17]. During the experimental period, hamsters were fed five different experimental diets for 4 weeks. Fat contributed to 30% of the total dietary energy and saturated fatty acids, monounsaturated fatty acids and polyunsaturated fatty acids contributed 16.8%, 8.5% and 4.7% of total dietary energy, respectively. The composition of the dietary fat resembled that of a typical Western diet. The experimental diets contained 0.08% (w/w) cholesterol. The composition of the mineral mix and the vitamin mix were described previously [18]. The detailed compositions of the experimental diets are shown in Table 3. These diets were designed to be identical in composition, except for the testing compounds. In order to ensure homogenous mixture of diets, cholesterol and PSE were incorporated into the fat blend and OA/UA were pre-mixed with a small amount of starch before they were mixed with other diet components.

#### 2.4.4. Sample Collection

In week 3, fecal samples were collected and weighted over two consecutive days. The feces were lyophilized and dry-weight of the fecal samples was recorded. Aliquots of homogenized fecal samples were used for measurements of fecal neutral sterols and bile acid excretion. At the end of the study, hamsters were fasted overnight (*∼*16 h). Blood samples were drawn into EDTA-wetted syringes by aorta puncture under halothane narcosis. The animals were killed by decapitation, and liver and intestinal samples were collected as described below. Plasma was collected after centrifugation at 1500 × g at room temperature for 10 min.

#### 2.4.5. Liver Sample Collection

 The liver was excised, flushed with PBS and liver weight was measured. About 1 g of liver tissue was sampled and frozen at −20°C for hepatic lipid analysis.

#### 2.4.6. Plasma Lipid and Lipoprotein Analysis

Plasma total cholesterol (TC) and TAG concentrations were determined enzymatically by using commercial assay kits (CHOD-PAP and GPO-PAP method, Roche Diagnostics, Basel, CH) and carried out on a COBAS Mira S automated analyzer (Roche Diagnostics, Basel, CH). Lipoprotein fractions were isolated from 0.6 mL plasma by sequential density-ultracentrifugation [19]. Three fractions were isolated based on the following densities: VLDL (*d* < 1.006 kg/L), LDL (1.006 < *d* < 1.055 kg/L), HDL (1.055 < *d* < 1.21 kg/L). TC and TAG concentrations in the lipoprotein fractions were determined as described above.

#### 2.4.7. Hepatic Cholesterol Analysis

A 300 mg portion of liver tissue was homogenized. Lipids in liver samples were extracted according to a modified method described by Bligh and Dyer [20], in which chloroform was substituted by dichloromethane. TC and TAG were determined as described above. Free cholesterol (FC) was measured using a commercial kit supplied by WAKO (Richmond, VA, USA). The concentration of cholesterol esters was calculated as the difference between TC and FC.

#### 2.4.8. Fecal Bile Acid and Neutral Sterol Measurements

Fecal bile acid concentrations were measured by GC and fecal neutral sterol concentrations were measured by GC–MS. The detailed procedures of these two assays have been described previously [21].

#### 2.4.9. Data Presentation and Statistical Analysis

All results are expressed as mean and standard error (SE). A two-way analysis of variance (ANOVA) (two-by-two table) was applied for determining the main effect of the hawthorn extract (i.e., hawthorn group plus hawthorn + PSE group compared with control group plus PSE group) and of the main effect PSE (i.e., PSE group plus hawthorn + PSE compared with control group plus hawthorn group). Statistical differences between the individual groups were calculated using a Dunnett multiple comparison test. The statistical analysis was conducted by using software SAS (version 9.1). Differences were considered significant at *P* < .05.

## 3. Results

### 3.1. Triterpenic Acid Content in Dried Hawthorn Fruit Powder and Hawthorn Extracts

**Figure 1:**
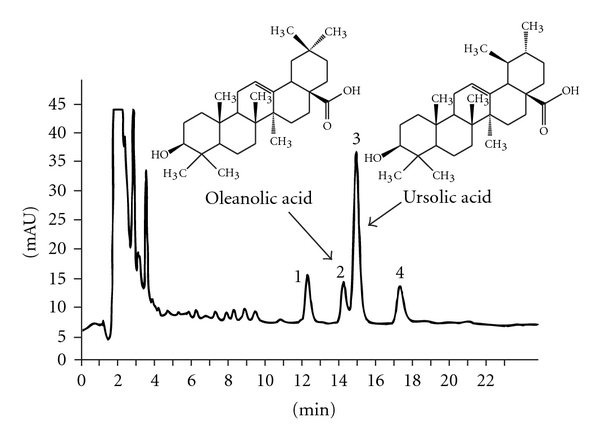
HPLC profile of the triterpenic acids in the dichloromethane extract of the dried hawthorn fruit powder is depicted. Dried hawthorn fruit powder was extracted sequentially with ethanol by using Soxhlet extractor at 80°C, followed by a dichloromethane extraction of the dried ethanol extract. Signal detection was carried out at 210 nm. Peak numbers: 1, (unknown); 2, OA; 3, UA; and 4, (unknown).

A typical HPLC profile of the triterpenic acids in the dichloromethane extract is shown in Figure 1. The triterpenic acid profile is comparable to that reported by others [13]. As shown in Table 1, heptane had only a limited capacity to extract triterpenic acids from the hawthorn powder. In contrast, dichloromethane extraction as compared to other tested solvents generated a higher purity (40.5%, w/w) of the triterpenic acids OA and UA in the extract. By using these solvents (except heptane) for extraction, it was calculated that the dried hawthorn fruit powder contained 0.24–0.38% (w/w) triterpenic acids (OA + UA). It was reported that fresh hawthorn (*C. pinnatifida*) fruit obtained from 37 cultivators contained 0.07–0.14% (average 0.11%) OA + UA [13].

For the animal study, dried hawthorn fruit powder was extracted with ethanol and was subsequently extracted with dichloromethane. Increasing the extraction temperature could increase the yield of dichloromethane extracted mass (9 g per 100 g hawthorn powder), while the triterpenic acid concentration in this extract was significantly decreased (3%). The calculated triterpenic acid concentration in the dried hawthorn powder, based on the extraction yield and OA + UA concentration in the dichloromethane extract, was 0.27%, which is in agreement with that (0.24%) in the extract carried out under room temperature (see above). The majority of the remainders in the hawthorn dichloromethane extract were mono-, and disaccharides based on GC–MS results (data not shown).

### 3.2. Study in Cultured Caco-2 Cells

#### 3.2.1. Validation of the In Vitro Test System

Cell viability was not influenced by any of the described incubations as indicated by the ATP assay (data not shown). Mevalonic acid (10 mM) increased isotope-labeled cholesteryl oleate production by up to 60%. An ACAT inhibitor (F-1394) inhibited ACAT activity by about 50% at a dose of 0.1 *μ*M. These data indicated that Caco-2 cells did respond to various stimuli by modulating cholesteryl ester (CE) production.

#### 3.2.2. Effect of Hawthorn Extracts on ACAT Activity (Table 2)

Caco-2 cells were incubated with increasing amounts of the hawthorn ethanol extract (10–300 *μ*g/mLmedium per well) for 24 h. The incorporation of [9,10-^3^H]oleic acid into cholesterol esters was dose-dependently inhibited (data not shown). To explore the potential bioactive compounds in hawthorn, extractions by using dichloromethane, ethylacetate, acetone, ethanol and heptane as solvents were made and the ACAT inhibitory effects of these extracts were tested in Caco-2 cells. The dichloromethane extract showed the strongest ACAT activity inhibitory effect amongst all tested extracts (Table 2). Via HPLC-analysis it was revealed that the contents in OA and UA were significantly different among these extracts, while the ratio of OA/UA was consistently 1 : 6 in all extracts. The ACAT inhibitory activity of these extracts was strongly associated with their OA and UA contents. To further confirm whether OA and UA are bioactive compounds, ACAT inhibitory effects of pure OA and UA were tested. Both OA and UA inhibited ACAT activity. The potency of the inhibitory effect of OA and UA was similar to that found for the extracts based on an equal amount of OA/UA. These *in vitro* data strongly suggested that OA and UA are responsible for the ACAT inhibitory effect of hawthorn. Compared to OA, UA had a slightly lower inhibitory activity. OA and UA had no effects on cell uptake of either [^14^C]cholesterol or [9,10-^3^H]oleic acid from the medium (data not shown), indicating that the inhibitory effect is via specifically inhibiting ACAT activity but not via inhibiting transport of precursors of cholesterol esters, that is, FC or fatty acids.

### 3.3. Hamster Study

#### 3.3.1. Food Intake and Body Weight Gain

Hamsters fed the different diets showed no significant differences in food intake (range 7–8.5 g/day for all animals) and body weight gain (5–5.5 g/week) during the 28-day treatment period.

#### 3.3.2. Plasma Lipids and Lipoproteins (Table 4)

Plasma VLDL and LDL fractions were combined as non-HDL. Hamsters administered hawthorn, PSE or hawthorn plus PSE had significantly lower plasma non-HDL cholesterol (non-HDL-C) concentrations than hamsters of the control group (*P* < .05). Only the hawthorn plus PSE diet lowered plasma TC as compared to control (*P* < .05). HDL-C and TAG concentrations were similar among all the five groups. OA/UA lowered plasma TC and non-HDL-C to an extent similar to that of hawthorn. However, this effect did not reach statistical significance (*P* < .30).

#### 3.3.3. Liver Weight and Hepatic Lipid Concentrations (Figure 2)

**Figure 2:**
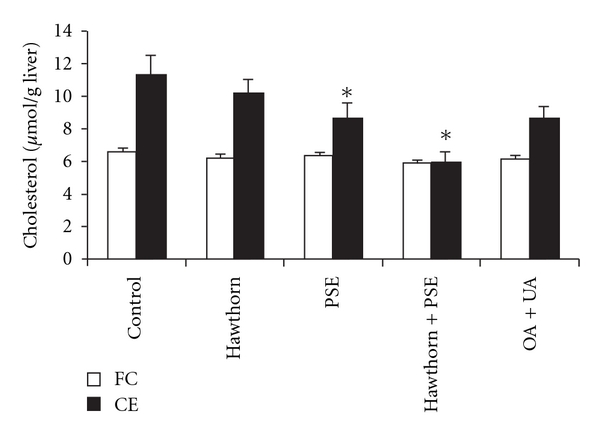
Hepatic FC and CE concentrations of hamsters fed the control and treatment diets for 4 weeks. A 300 mg portion of liver tissue from each animal was homogenized. Lipids were extracted using the method described by Bligh and Dyer and total cholesterol and FC were measured enzymatically. Concentration of CE was calculated as the difference between total cholesterol and FC. Data are presented as mean ± SE (*n* = 20 animals per group). *P < .01 as compared to control.

At the end of the study, absolute (range 4.2–4.4 g) and relative liver weights (range 3.5–3.6% of body weight) did not differ between the groups. Hepatic FC concentrations were not significantly different between the groups (*P* = .24). However, hawthorn extract, PSE and OA/UA lowered hepatic concentration of cholesterol esters by 9% (*P* = .06), 23% (*P* < .01) and 22% (*P* = .05), respectively (Figure 2). The combination of hawthorn extract plus PSE resulted in the strongest decrease (46%) in hepatic cholesterol esters (*P* < .01).

#### 3.3.4. Fecal Excretion of Neutral Sterols and Bile Acids (Table 5)

Hawthorn extract, PSE or OA/UA did not change daily fecal dry weight. Fecal output of neutral sterols was significantly increased in hamsters fed the hawthorn extract or OA/UA compared to the control diet. The PSE diet slightly (3%) increased fecal neutral sterol excretion but this did not reach statistical significance (*P* > .05). Diets containing hawthorn or OA/UA significantly decreased fecal output of total bile acids (*P* < .05).

The two PSE-containing diets (PSE and hawthorn plus PSE) significantly increased fecal plant sterol excretion by 7.5-fold (*P* < .01) while with hawthorn and OA/UA added to the diets the fecal plant sterol excretion was not different as compared to the control diet.

## 4. Discussion

This study investigated the effect of hawthorn on cholesterol metabolism in hamsters and in human Caco-2 cells. The dichloromethane hawthorn extract lowered plasma non-HDL-C by 8% without changing HDL-C. This finding is further supported by the fact that the hawthorn extract also reduced hepatic total cholesterol and CE contents. Hepatic cholesterol esters are the storage form of cholesterol in the liver. In rodents such as mice [22] and hamsters [21], hepatic CE concentration is more sensitive than plasma cholesterol in reflecting to the cholesterol lowering effect of dietary ingredients. Besides in hamsters, hawthorn (in the form of extracts) has been reported to lower plasma cholesterol concentration in rats [4, 5] and rabbits [2]. These results, taken together, provide scientific evidence that hawthorn could be a useful natural ingredient for lowering plasma cholesterol concentrations in humans [6–8]. Furthermore, our study showed that the combination of hawthorn extract with PSE had an additive cholesterol lowering effect.

Hawthorn is rich in triterpenic acids (OA and UA) and polyphenols (e.g., epicatechin, procyanidin B2, procyanidin B5, procyanidin C1, hyperoside, isoquercitrin and chlorogenic acid) [13]. The present study demonstrated that in particular OA and UA are responsible for the cholesterol lowering effect of hawthorn. This concept is based on the fact that the mixture of OA and UA, which resembled the amount of these compounds in the hawthorn extract, exerted effects to a similar magnitude as observed with the hawthorn extract both *in vitro* and *in vivo*. These effects include (i) inhibition of ACAT activity (*in vitro*), (ii) a decrease of plasma and liver cholesterol concentrations to a similar extent, (iii) an increase in fecal neutral sterol excretion and (iv) a reduction of fecal bile acid excretion. Although we did not test the effect of hawthorn polyphenols on their ACAT inhibitory activity in this study, we have found in our vitro experiments that other natural compounds such as flavonoids, for example, hesperetin, genistein, or flavanolignan (e.g., silymarin) and sterols did not inhibit ACAT activity in Caco-2 cells (data not shown).

Several lines of evidence show that OA and UA lower plasma cholesterol via inhibiting ACAT activity. First, our cellular experiments showed that the ACAT inhibitory effect of the hawthorn extracts correlates with the triterpenic acid (OA plus UA) content in the extracts. Secondly, the pure OA and UA exerted their ACAT inhibitory effect in Caco-2 cells to an extent similar to that of hawthorn extracts containing equal amounts of OA and UA. Thirdly, both the hawthorn extract and OA/UA mix increased fecal neutral sterols excretion in hamsters, indicating decreased intestinal cholesterol absorption. Compared to PSE (0.24% of diet, w/w), a much lower dose of OA/UA (0.01% of diet, in pure form or derived from hawthorn extract) caused a similar cholesterol lowering effect. These results suggest that the cholesterol lowering mechanism by which hawthorn and OA/UA lower plasma cholesterol is different from that of PSE. PSE lower plasma cholesterol via partly inhibiting intestinal cholesterol absorption, primarily via competing with cholesterol for incorporation into dietary mixed micelles [14]. Therefore a relatively large amount of PSE (0.24% of diet, which is more than the dietary cholesterol content (0.08%) in this study) is needed to competitively inhibit cholesterol absorption. For OA/UA, which inhibits cholesterol absorption at a low dose (0.01%), a plausible explanation would be that OA/UA reduced cholesterol absorption via primary inhibition of intestinal ACAT activity. Our findings are further supported by a recent study performed by Lee et al. [23] in which both OA and UA isolated from the leaves of *Lycopus lucidus* inhibited ACAT activity in Hi5 cells transfected with human ACAT genes. Intestinal ACAT plays an important role in the absorption of cholesterol. In enterocytes, absorbed cholesterol is esterified by ACAT to form cholesterol esters, which serve as part of the core lipid for chylomicron assembly. Several synthetic ACAT inhibitors have been shown to lower plasma cholesterol in animals [24, 25].

Hawthorn and OA/UA lowered hepatic CE concentrations. This CE lowering effect of hawthorn may not be attributed to inhibiting ACAT activity in the liver because both PSE and hawthorn had similar effects on hepatic CE concentration. It has been established that PSE are poorly absorbed from gut lumen and do not modulate hepatic ACAT activity [22]. Thus, the remarkable decrease in hepatic cholesterol esters with both hawthorn and PSE is more likely the result of their plasma cholesterol lowering effect via reducing cholesterol absorption. Therefore, the decreased amount of hepatic cholesterol esters is a secondary (consequential) effect rather than a primary effect. Further supporting this concept is the fact that hepatic FC concentration was not increased by the treatment of hawthorn or OA/UA. Moreover, a synthetic ACAT inhibitor (YIC-C8-434) was also shown to lower plasma cholesterol concentration in rats via inhibiting intestinal, but not hepatic ACAT activity [26]. Hawthorn and OA/UA slightly, but statistically significantly, lowered fecal bile acid output in hamsters. In contrast to these results, Zhang et al. [2, 3] demonstrated that hawthorn could enhance fecal bile acid excretion by up-regulation of hepatic cholesterol 7-alpha-hydroxylase activity in hamsters [3] and rabbits [2]. Since cholesterol is the precursor of bile acids, it can not be excluded that the decreased fecal bile acid excretion by hawthorn or OA/UA in this study is a consequence of the hepatic cholesterol lowering effect of these ingredients. Alternatively, Zhang et al. used an ethanol extract of hawthorn [3] or hawthorn powder [2] while in this study we used a dichloromethane extract of dried hawthorn fruit powder. Based on our findings, the purity of the triterpenic acids in the dichloromethane extract was higher than that in ethanol extract (Table 1). Possibly, other ingredients in the ethanol extracts or in the hawthorn powder used by Zhang et al. [2, 3] may have increased fecal bile acid excretion. Further studies are warranted to fully understand the effect of hawthorn and OA/UA on hepatic bile acid metabolism.

There are two types of ACAT, that is, ACAT1 and ACAT2, in the body [27]. The ACAT1 gene is expressed in almost all tissues [27]. ACAT1 is not involved in lipoprotein formation. A selective ACAT1 inhibitor (K604) showed no cholesterol lowering effect [28]. Similarly, knockout of the ACAT1 gene did not lower plasma cholesterol concentrations in mice but rather increased plasma cholesterol [29]. In contrast, ACAT2 is expressed only in hepatocytes and enterocytes and its main function is to provide cholesterol esters for transport in lipoproteins [27]. ACAT2-knockout mice, compared to wild-type mice, absorbed less cholesterol [30] and they were resistant to diet-induced hypercholesterolemia [31]. In this context, the cholesterol lowering effect of hawthorn and OA/UA seems more likely a result of inhibiting intestinal ACAT2 activity.

In conclusion, the present hamster study shows that hawthorn exerts a cholesterol lowering effect via a mechanism which is different from that of PSE. Hawthorn and PSE had an additive effect on lowering plasma cholesterol concentrations and could therefore offer a promising combination for further cholesterol-lowering by dietary means. OA and UA are the bioactive compounds responsible for the beneficial effects of hawthorn, which reduce intestinal cholesterol absorption via inhibition of the intestinal ACAT activity.

## Figures and Tables

**Table 1:** OA and UA concentrations in the dried hawthorn fruit powder and extracts.

Extraction solvents	Hawthorn powder (g)	Dry extract yield (g)	OA and UA in dry extracts (g/100 g)			Calculated OA + UA in hawthorn powder (g/100 g)
			OA	UA	OA + UA	
	I	II			III	(II × III)/I
Heptane	20^a^	0.044	1.0	1.6	2.6	0.01
Ethanol	20	0.986	1.5	6.2	7.7	0.38
Aceton	20	0.260	5.4	20.3	25.7	0.33
Ethylacetate	20	0.149	8.1	30.8	38.9	0.29
Dichloromethane	20	0.118	9.0	31.5	40.5	0.24
Dichloromethane	425^b^	38.25	0.4	2.6	3.0	0.27

^a^ For the *in vitro* experiments, 20 g of dried hawthorn powder was extracted with 100 mL dichloromethane, ethylacetate, acetone, ethanol or heptane, respectively, at room temperature for 1.5 h. The obtained extracts were then dried under nitrogen flow.

^b^ For the animal study, 425 g dried hawthorn fruit powder was extracted with ethanol followed by dichloromethane (details are described in Section 2). Triterpenic acid concentrations in the extracts were measured by HPLC and data are presented as mean value of two measurements.

**Table 2:** ACAT inhibitory effect of hawthorn extracts and pure OA and UA in Caco-2 cells.

Test materials	Final OA + UA content in medium (*μ*M)	ACAT activity (percentage of control)
Control	0	100 ± 0.0

Solvent extract (50 *μ*g/well)
Heptane	2.8	77.2 ± 11.8
Ethanol	8.4	86.4 ± 10.8
Acetone	28.1	46.7 ± 8.6
Ethylacetate	42.6	36.5 ± 8.0
Dichloromethane	44.3	37.9 ± 11.1

Pure compounds
OA	50.0	30.9 ± 3.9
UA	50.0	50.5 ± 8.5

Hawthorn powder was extracted with indicated solvents to generate various hawthorn extracts. Caco-2 cells were incubated with medium (1 mL) containing 50 *μ*g of each hawthorn extract dissolved in 10  *μ*L DMSO or indicated amount of OA or OA for 24 h. OA and UA content in each extract is presented in Table 1. 4 h before the end of the incubation, [9,10-^3^H]oleic acid (62.5 *μ*M, 1 *μ*Ci/well) was added to each well to monitor cholesterol ester synthesis. Cellular lipids were extracted by solvents and cholesterol esters were isolated using a silica column as described in Methods. The radioactivity of generated [^3^H]cholesterol esters was measured by scintillation counting. Data are presented as mean ± SE from six independent incubations. ACAT activity in each treatment was significantly different from control value (*P* < .05).

**Table 3:** Composition of the control and treatment diets.

Ingredients (g/kg diet)	Diets				
	Control	Hawthorn	PSE	Hawthorn + PSE	OA + UA
Calcium caseinate	161.4	161.4	161.4	161.4	161.4
Plant sterol esters (PSE)	—	—	2.4	2.4	—
Dichloromethane extract of hawthorn	—	3.7	—	3.7	—
Oleanolic acid (OA)	—	—	—	—	0.015
Ursolic acid (UA)	—	—	—	—	0.095
Vitamin mix	11.3	11.3	11.3	11.3	11.3
Mineral mix	39.7	39.7	39.7	39.7	39.7
Arbocel (fibres)	56.7	56.7	56.7	56.7	56.7
Fat	126.1	126.1	126.1	126.1	126.1
l-Cysteine hydrochloride	2.1	2.1	2.1	2.1	2.1
Choline bitartrate	2.8	2.8	2.8	2.8	2.8
Cholesterol	0.8	0.8	0.8	0.8	0.8
Maize starch	599.1	595.4	596.7	592.9	599.0

**Table 4:** Plasma and lipoprotein lipid concentrations (mmol/L) of hamsters fed the control and treatment diets.

Plasma lipids	Diets				
	Control	Hawthorn	PSE	Hawthorn + PSE	OA + UA
TC	5.85 ± 0.21	5.84 ± 0.19 (0%)	5.56 ± 0.19 (−5%)	4.93 ± 0.19 (−16%)*	5.39 ± 0.18 (−8%)
Non-HDL-C	2.08 ± 0.12	1.92 ± 0.10 (−8%)*	1.90 ± 0.08 (−9%)*	1.64 ± 0.10 (−21%)*	1.96 ± 0.08 (−6%)
HDL-C	3.51 ± 0.14	3.53 ± 0.14 (+1%)	3.40 ± 0.14 (−3%)	3.18 ± 0.12 (−9%)	3.27 ± 0.15 (−7%)
TAG	3.79 ± 0.40	3.56 ± 0.40 (−6%)	3.45 ± 0.35 (−9%)	3.19 ± 0.23 (−16%)	3.37 ± 0.36 (−11%)

Hamsters were fed diets as indicated in Table 3 for 4 weeks. At the end of the study, fasting (16 h) blood samples were collected and plasma total cholesterol (TC) and triacylglycerol (TAG) concentrations were determined enzymatically. Plasma lipoproteins (VLDL, LDL and HDL) were fractionated by density-ultracentrifugation. Cholesterol (-C) concentrations in HDL and non-HDL (VLDL + LDL) were measured enzymatically. Data are presented as means ± SE (*n* = 20 animals per group). Data in parentheses represent percentage difference compared to control group. Asterisk indicates that the value is significantly different from that of control (*P* < .05).

**Table 5:** Fecal output and fecal excretion of plant sterols, neutral sterols and bile acids in hamsters fed the control and treatment diets.

Fecal	Diets				
	Control	Hawthorn	PSE	Hawthorn + PSE	OA + UA
Fecal output (g/day)					
Dry weight	0.63 ± 0.03	0.62 ± 0.03	0.57 ± 0.03	0.61 ± 0.03	0.63 ± 0.02
Fecal excretion of sterols and bile acids (*μ*mol/day)					
Plant sterols	2.19 ± 0.07	2.63 ± 0.17 (+20%)	16.35 ± 1.13 (+647%)**	17.86 ± 1.09 (+716%)**	2.40 ± 0.10 (+9%)
Neutral sterols	9.90 ± 0.43	11.54 ± 0.54 (+17%)*	10.24 ± 0.57 (+3%)	11.58 ± 0.78 (+17%)*	12.14 ± 0.46 (+23%)*
Bile acids	1.68 ± 0.13	1.42 ± 0.14 (−15%)*	1.64 ± 0.16 (−2%)	1.31 ± 0.10 (−22%)*	1.30 ± 0.11 (−23%)*
Neutral sterols + Bile acids	11.71 ± 0.47	13.04 ± 0.61 (+11%)	11.97 ± 0.66 (+2%)	12.85 ± 0.76 (+10%)	13.47 ± 0.49 (+15%)

PSE: plant sterol esters. Forty-eight hours fecal samples were collected in Week 3. Plant sterols include campesterol, campestanol, stigmasterol, stigmastanol, beta-sitosterol, sitostanol and brassicasterol. Neutral sterols include cholesterol, cholestanol and coprostanone. Values are means ± SEM (*n* = 20 animals per group). Data in parenthesis represent percentage difference compared to control group.

*P < .05; **P < .01.
